# Different Photodissociation Mechanisms in Fe(CO)_5_ and Cr(CO)_6_ Evidenced with Femtosecond Valence Photoelectron Spectroscopy and Excited-State Molecular Dynamics Simulations

**DOI:** 10.1021/acs.jpclett.4c02025

**Published:** 2024-11-20

**Authors:** Henning Schröder, Michael R. Coates, Raphael M. Jay, Ambar Banerjee, Nomi L.A.N. Sorgenfrei, Christian Weniger, Rolf Mitzner, Alexander Föhlisch, Michael Odelius, Philippe Wernet

**Affiliations:** ● Institut für Physik und Astronomie, Universität Potsdam, Haus 28 Karl-Liebknecht-Straße 24/25, 14476 Potsdam-Golm, Germany; ◧ Helmholtz-Zentrum Berlin für Materialien und Energie, Hahn-Meitner-Platz 1, 14109 Berlin, Germany; § Department of Physics, Stockholm University, AlbaNova University Center, SE-106 91 Stockholm, Sweden; ∥ Department of Physics and Astronomy, Uppsala University, Box 516, SE-751 20 Uppsala, Sweden

## Abstract

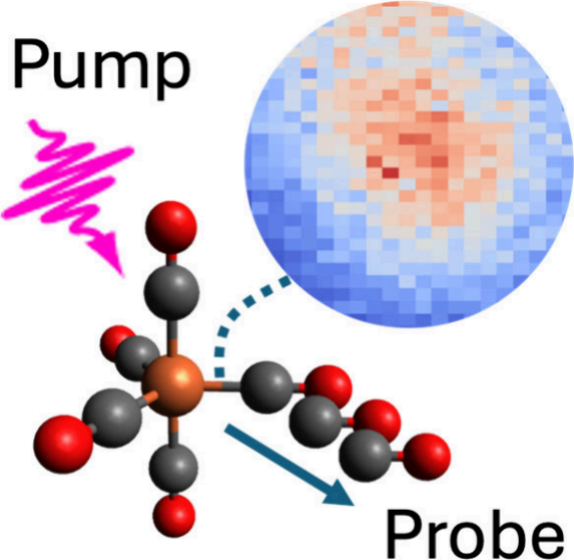

Measured and calculated time-resolved photoelectron spectra and excited-state molecular dynamics simulations of photoexcited gas-phase molecules Fe(CO)_5_ and Cr(CO)_6_ are presented. Samples were excited with 266 nm pump pulses and probed with 23 eV photons from a femtosecond high-order harmonic generation source. Photoelectron intensities are seen to blue-shift as a function of time from binding energies characteristic of bound electronic excited states via dissociated-state energies toward the energies of the dissociated species for both Fe(CO)_5_ and Cr(CO)_6_, but differences are apparent. The excited-state and dissociation dynamics are found to be faster in Cr(CO)_6_ because the repopulation from bound excited to dissociative excited states is faster. This may be due to stronger coupling between bound and dissociative states in Cr(CO)_6_, a notion supported by the observation that the manifolds of bound and dissociative states overlap in a narrow energy range in this system.

The dissociation dynamics of metal–carbonyl bonds has attracted attention for decades.^1−7^ This is due to the importance of understanding metal–carbonyl dissociation and the preceding excited-state relaxation dynamics for understanding C–H activation by metal complexes,^8−10^ Fe–CO dissociation in biomolecules,^6^ or fundamentals of metal–carbonyl bonding and dynamics.^2,4^ 3d transition-metal carbonyl complexes have ever since served as ideal model systems for mechanistic insight into metal–carbonyl excited-state and dissociation dynamics.^2,3^ A generally accepted mechanism has emerged according to which initial excitation of the complex to an optically bright metal-to-ligand charge-transfer (MLCT) state is thought to be followed by excited-state relaxation dynamics in a manifold of MLCT states with subsequent dissociation via metal-centered (MC) states (with, in the gas phase, potential further dissociation of more carbonyls).^11^ The direct population of the MC states is not possible due to symmetry rules in both Fe(CO)_5_^7^ and Cr(CO)_6_.^12^ While this is a useful first approximation that is often also used to describe the dissociation of metal–carbonyl complexes in condensed-phased photochemistry,^13,14^ it neglects potential differences between different metal carbonyls due to differences in bonding and structure. In a seminal series of investigations, Werner Fuß and colleagues have studied and compared the dissociation dynamics of different gas-phase metal–carbonyl complexes with femtosecond ionization spectroscopy.^5,15−17^ The difference that they found between Fe(CO)_5_ and Cr(CO)_6_ is particularly noteworthy. While it was found that dissociation in Fe(CO)_5_ is slower than in Cr(CO)_6_,^5^ relations to bonding, excited-state populations, and structure or symmetry of the complexes could not be inferred. We have recently begun revisiting the femtosecond photochemistry of some of the metal–carbonyl complexes in the gas phase with electronic-structure spectroscopy measurements using femtosecond X-ray and extended UV (XUV) pulses^11^ or with excited-state molecular dynamics (MD) dynamics simulations.^7^ Here, and for the first time with a combination of methods used previously, namely, time-resolved XUV photoelectron spectroscopy experiments^18−23^ and excited-state MD simulations^24^ with spectrum calculations,^25^ we address the photochemistry of gaseous Fe(CO)_5_ and Cr(CO)_6_ to probe in which way differences in symmetry and excited-state populations determine the dissociation dynamics. With a new observable from femtosecond-resolved valence photoelectron spectroscopy combined with excited-state MD simulations we aim at rationalizing the different time scales of CO dissociation in Fe(CO)_5_ and Cr(CO)_6_.

We show in Figure 1 the measured steady-state and time-resolved valence photoelectron spectra of Fe(CO)_5_ and Cr(CO)_6_ in the gas phase. The time-resolved spectra were measured in a pump–probe scheme with pump pulses photoexciting the samples at 266 nm and XUV pulses with a photon energy of 23 eV from a femtosecond high-order harmonic generation source for probing. The measurements were done with a modified version of a set up described elsewhere,^26^ and details of the present experiments are given in the Supporting Information. With the temporal resolution of 260 fs achieved here (Gaussian full width at half-maximum, fwhm, of the cross correlation of pump and probe pulses as mainly determined by the duration of the pump pulses) it is important to note that we do not have sufficiently high temporal resolution to test the coherent oscillations and periodic bursts of CO release predicted in our earlier theoretical study.^7^ We instead focused on investigating the differences in excited-state dynamics and dissociation times between Fe(CO)_5_ and Cr(CO)_6_.

**Figure 1:**
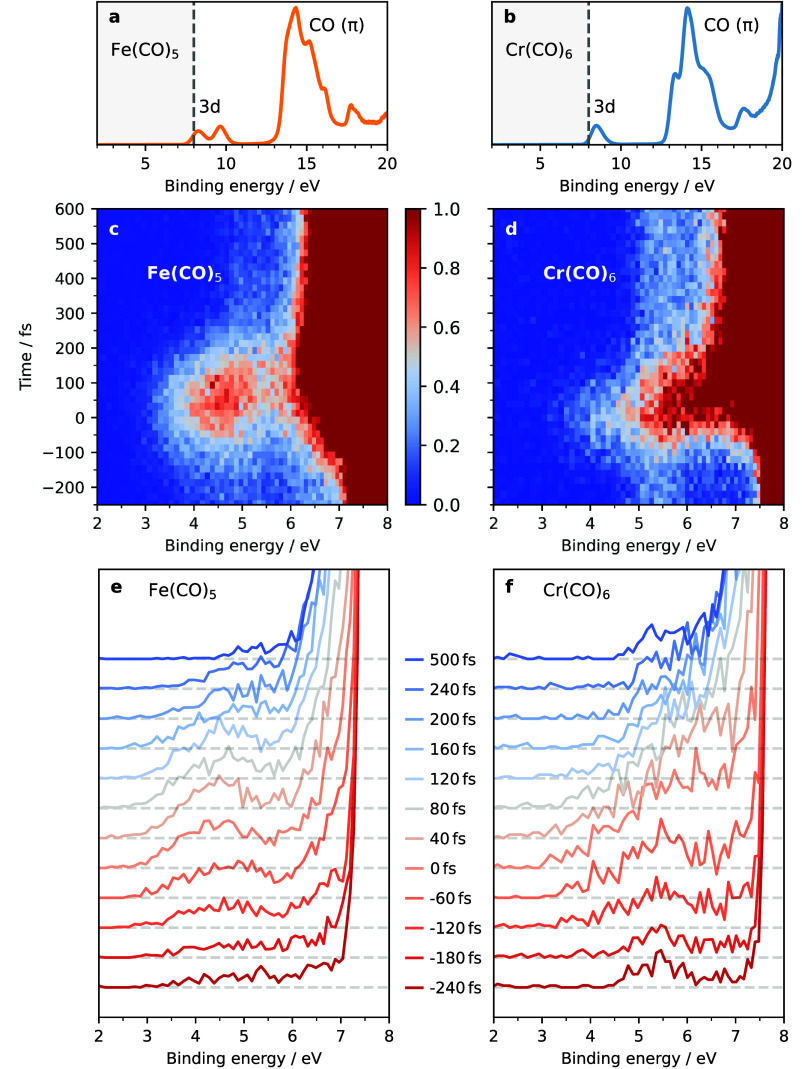
Measured steady-state and time-resolved valence photoelectron spectra of Fe(CO)_5_ and Cr(CO)_6_ in the gas phase. (a and b) Steady-state spectra of ground-state Fe(CO)_5_ and Cr(CO)_6_ with assigned metal 3d peaks (photoionization from metal 3d orbitals) and CO π peaks (photoionization from CO π orbitals). (c and d) Time-resolved pump–probe photoelectron intensities of Fe(CO)_5_ and Cr(CO)_6_ following photoexcitation at 266 nm as a function of pump–probe time delay and up to binding energies of 8 eV (just below the metal 3d peaks of the ground-state species, intensities encoded in color according to the given color bar, maximum intensities normalized to one by dividing intensities by 1 × 10^3^ for Fe(CO)_5_ and by 1 × 10^4^ for Cr(CO)_6_, photoelectron intensities at highest binding energies are saturated in this representation). (e and f) Photoelectron spectra of Fe(CO)_5_ and Cr(CO)_6_ at the given time delays (relative intensities are as measured, absolute intensities are in arbitrary units, spectra correspond to horizontal cuts through the maps in panels c and d).

The steady-state valence photoelectron spectrum of ground-state Fe(CO)_5_ in Figure 1a exhibits two peaks at 8.2 and 9.5 eV, corresponding to photoionization from the degenerate Fe 3d (e′) and Fe 3d (e*″*) metal-centered orbitals, respectively.^11,27^ For Cr(CO)_6_ (Figure 1b) there is a single peak at 8.4 eV corresponding to photoionization from the triply degenerate Cr 3d (t_2g_) orbitals.^28−32^ In both systems, the manifold of peaks centered around 15 eV is associated with photoionization from CO (π and σ) orbitals.^29,31^ The steady-state spectra indicate the interesting binding energy region for closer inspection by time-resolved spectroscopy. With electronic excitation, we expect new peaks to arise below the Fe/Cr 3d peaks of ground-state Fe(CO)_5_ and Cr(CO)_6_ (below the dashed lines in Figure 1a,b) due to photoionization of the excited “active” electrons (electrons in the occupied orbitals with highest energies). The aim is to use these signals to characterize the excited-state and dissociation dynamics.

The time-resolved valence photoelectron spectra of photoexcited Fe(CO)_5_ and Cr(CO)_6_ in Figure 1c,d verify our expectation with new peaks arising at binding energies of 3–7 eV and within 300 fs after excitation. Note that the low-energy parts of these features may partly overlap with transient intensities arising from so-called side bands (ref (33) and references therein; simultaneous absorption of pump and probe pulses generating peaks that are indistinguishable from excited-state intensities). Side-bands are expected around pump–probe delay times of 0 fs and at binding energies of the lowest ground-state 3d peaks minus the pump energy (266 nm corresponding to 4.7 eV) and hence at 3.5 eV in Fe(CO)_5_ (8.2 eV – 4.7 eV = 3.5 eV) and at 3.7 eV in Cr(CO)_6_ (8.4 eV – 4.7 eV = 3.7 eV). As is clear from Figure 1c, intensity accumulates for Fe(CO)_5_ in a region around 4–5 eV for times 0–100 fs and hence is clearly offset to the expected sideband intensities at 3.5 eV/0 fs. In Cr(CO)_6_ as well, a sideband peak is not observed at 3.7 eV/0 fs but rather continuously growing in intensities. An additional complication in our experiment masks the targeted dynamics. Spectra of Cr(CO)_6_ at −240 and 500 fs in Figure 1f show that part of the intensity at 5–6 eV is time-independent (part of the intensity in this region does not change). This is also visible in Figure 1d as a faint light-blue vertical intensity band underlying the data for all times at 5–6 eV. This time-independent intensity portion originates from photoionization of ground-state Cr(CO)_6_ by another harmonic than the main selected one at 23 eV due to “spillover” in our monochromator (light of another photon energy than the nominally selected harmonic ionized Cr(CO)_6_). These spurious intensities are more apparent in Cr(CO)_6_ compared to Fe(CO)_5_ since overall pump–probe intensities are much smaller in Cr compared to Fe.

Our data in Figure 1c,d with detailed views in Figure 1e,f clearly reflect differences in the photodissociation mechanisms of Fe(CO)_5_ and Cr(CO)_6_. In Fe(CO)_5_ (Figure 1e) a peak at 4–5 eV starts building up at ∼120 fs with maximum intensity around 80 fs, indicative of a transient excited-state population (a sideband potentially contributes to the lower-binding energy flank of this peak, and due to the temporal resolution of 260 fs fwhm the dynamics occurs prior to delay time 0 fs). This transient intensity decreases and shifts to higher binding energies within 100–200 fs, indicating repopulation in the excited-state manifold. Ultimately, a strong band grows at 6–7 eV corresponding to dissociated species.^11^ In Cr(CO)_6_, in contrast (Figure 1f), we see much weaker intensity accumulations here at 5–6 eV and with a maximum around or shortly after 0 fs. This indicates that the excited-state dynamics of Cr(CO)_6_ are faster than those in Fe(CO)_5_ with the system proceeding faster to dissociated species (sideband intensities centered at 0 fs potentially contribute to the lower-binding energy flank). This transient intensity then decreases and shifts to higher binding energy with a strong rise in a band at 6.5–7.5 eV characteristic of the dissociated species. We note that previous literature has shown that both Fe(CO)_5_ and Cr(CO)_6_ dissociate with a quantum yield approaching unity (Φ ≈ 1). Hence, all time-dependent changes in our photoelectron spectra reflect the coupling of the excited state and dissociation dynamics.

We illustrate the differences between Fe(CO)_5_ and Cr(CO)_6_ with the measured photoelectron intensities plotted as a function of pump–probe delay time (delay traces) and the calculated populations of species shown in Figure 2. Because the measured delay traces include photoelectron intensities below 7 eV, they are dominated by excited-state information. For both Fe(CO)_5_ and Cr(CO)_6_, we find in experiment (Figure 2a,b) that the higher the binding energy of a probed feature, the later it reaches its maximum intensity. This quantifies how photoelectron intensities blue-shift with increasing time, a phenomenon observed before.^34^

**Figure 2:**
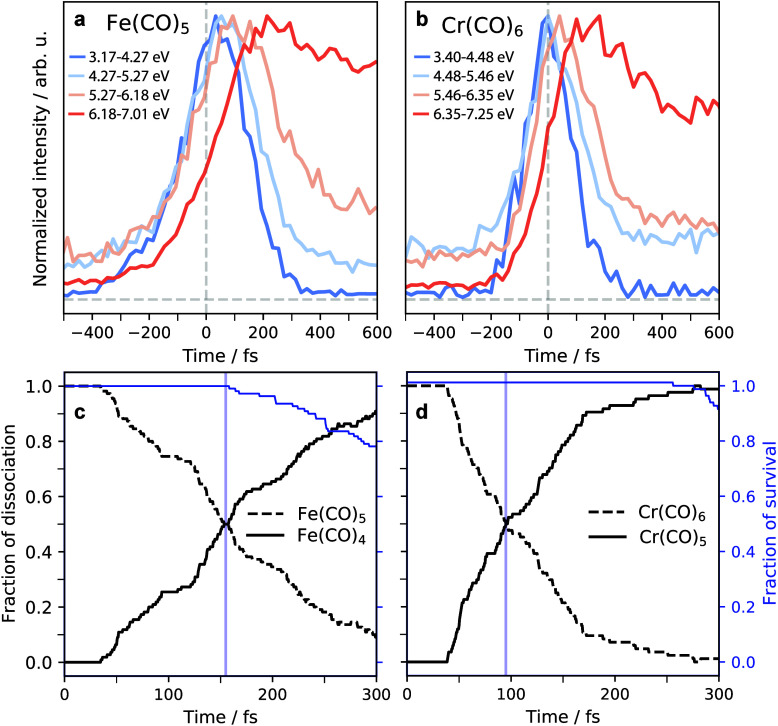
Measured photoelectron intensities and calculated populations as a function of pump–probe time delay. (a and b) Delay traces from measured photoelectron intensities for Fe(CO)_5_ and Cr(CO)_6_ plotted as a function of time delay and as integrated for the given binding-energy intervals (horizontal dashed lines indicate zero photoelectron intensities, intensities of different delay traces are normalized to the same maximum, traces correspond to vertical cuts through the maps in Figure 1c,d around the given binding energies). (c and d) Population fractions of Fe(CO)_5_, Fe(CO)_4_, Cr(CO)_6_, and Cr(CO)_5_, calculated from the excited-state molecular dynamics simulations, and trajectory survival as a function of time delay after photoexcitation where the populations of Fe(CO)_4_ and Cr(CO)_5_ represent the fractions of dissociation (dissociation fractions of 50% are indicated with blue vertical lines).

Notably, when comparing the delay traces of Fe(CO)_5_ and Cr(CO)_6_ taken for the same binding energy intervals with respect to the respective ground-state photoelectron peaks in Figure 2a,b (the intervals of the two systems differ by the same amount as their ground-state peaks), we can quantify the “slower dynamics” in Fe(CO)_5_ by the observation that the times when the delay traces reach their maximum intensities are shifted to later times in Fe(CO)_5_ compared to Cr(CO)_6_. In Fe(CO)_5_, all extracted delay traces are shifted with respect to 0 fs because excited states with the given binding energies are populated considerably after 0 fs. All traces are asymmetric with tails to longer times, presumably because excited states decay slower than they grow in. The prominent 4–5 eV excited-state peak, in particular, reaches its maximum at 80 fs, indicating considerable excited-state population at this time. By contrast, in Cr(CO)_6_ the delay traces at both 3.4–4.5 and 4.5–5.5 eV are centered around 0 fs without or with small asymmetries, presumably because excited states are populated and decay within the temporal resolution of our experiment. Traces taken at higher binding energies are shifted to later times, and they are asymmetric, albeit with less asymmetry than in Fe(CO)_5_. Our experimental observation of “slower dynamics” in Fe(CO)_5_ compared to Cr(CO)_6_ with femtosecond photoelectron spectroscopy confirms the femtosecond ionization spectroscopy results from Fuß et al.^15,17^ With our excited-state molecular dynamics simulations and photoelectron spectrum calculations, we now proceed to explain this difference.

We previously established for dissociating Fe(CO)_5_ in our analysis of simulated synchronous oscillations with 90 fs intervals and periodic bursts of CO release^7^ a criterion for dissociation by inspecting the value of the lowest Fe–C bond length attained only once by each simulated trajectory prior to dissociation. This corresponds to an Fe–C bond length of 2.5 Å. In this study, we extended this analysis to Cr(CO)_6_ with additional excited-state MD simulations and found similar synchronous oscillations and periodic bursts of CO release. We also found that 2.6 Å was the shortest Cr–C bond length that was crossed only once by all dissociating trajectories. Interestingly, also the ground-state Cr–C bond lengths are on average 0.1 Å longer than the ground-state Fe–C bond lengths, and hence, the overall maxima of the Fe–C and Cr–C oscillations occur at the same elongation relative to the equilibrium distance.

We applied these arbitrary measures of dissociation to all singly dissociating trajectories for Fe(CO)_5_ and Cr(CO)_6_ to determine the fractions of Fe(CO)_5_/Fe(CO)_4_ and Cr(CO)_6_/Cr(CO)_5_ as a function of time (plotted in Figure 2c,d). We additionally plot a “Fraction of Survival” in Figure 2c,d, which is a measure of the number of active trajectories at each time step. This is because many trajectories do not survive after dissociation due to problems of SCF convergence in the underlying DFT calculations. Still, the formation of Fe(CO)_4_ in bursts is discernible in the traces in Figure 2c in the form of steps at around 50, 130, and 200 fs. Additional simulation results presented below show that Cr(CO)_5_ also forms in bursts despite the fact that steps are not discernible in the traces in Figure 2d. These additional simulations will also be used to explain the common initial delay of 30 fs in the formation of both Fe(CO)_4_ and Cr(CO)_5_ (see Figure 2c,d). The measures of dissociation introduced above were also used to define an arbitrary or approximate “dissociation time”. This “dissociation time” is given here by the time when the ensembles have reached a 50% fraction of dissociated Fe(CO)_4_ and Cr(CO)_5_ species (the minor fraction of trajectories where multiple COs dissociate is not included in the analysis). In agreement with our experimental observation of “slower dynamics” in Fe(CO)_5_, we find approximate “dissociation times” of 155 fs in Fe(CO)_5_ and 95 fs in Cr(CO)_6_ (blue lines in Figure 2c,d).

In the following, we investigate the conversion of Fe(CO)_5_ and Cr(CO)_6_ from the initial MLCT states to the lower MC states where the dissociation occurs, how the simulated difference in “dissociation time” between the two systems results from the underlying “slower dynamics” in excited-state repopulations in Fe(CO)_5_, and how this relates to our experimental observations. We start by illustrating when and from which states the dissociation occurs by plotting in Figure 3a,b the Fe–C and Cr–C distances to the dissociating CO as a function of time for each singly dissociating trajectory.

**Figure 3:**
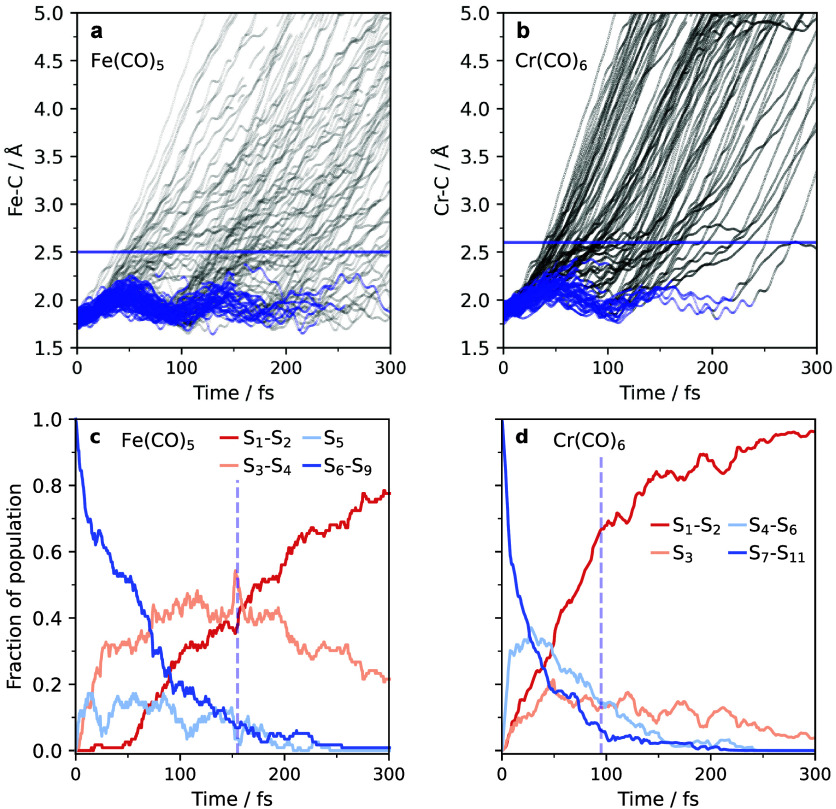
Calculated excited-state dynamics of Fe(CO)_5_ and Cr(CO)_6_. (a and b) Calculated Fe–C and Cr–C trajectories as a function of time delay after excitation; trajectories in bound and dissociative states are plotted blue and black, respectively (blue horizontal lines indicate distances above which the metal–carbonyl bonds can be regarded as dissociated. (c and d) Calculated population fractions of excited states of Fe(CO)_5_ and Cr(CO)_6_ as a function of time delay after excitation for bound (blue) and dissociative states (red), the blue lines indicate dissociation fractions of 50% as taken from Figure 2c,d (populations of different excited states are summed or plotted as indicated according to their common qualitative behavior as a function of time).

The trajectories clearly exhibit an intricate interplay of bond-length oscillations and dissociation events within 300 fs of excitation for both Fe(CO)_5_ and Cr(CO)_6_. The horizontal blue lines in Figure 3a,b indicate the distance criteria used in Figure 2 to define “dissociation” thereby demarking excited-state dynamics with oscillations in bound states (trajectories below the blue lines) and escapes from the bound regions on dissociative states (trajectories above the blue lines). Dissociated metal–CO distances, according to the analogous distance criteria, appear approximately 30 fs after photoexcitation at the earliest for both Fe(CO)_5_ and Cr(CO)_6_. The corresponding common initial delay in the formation of Fe(CO)_4_ and Cr(CO)_5_ (Figure 2c,d) can hence be explained by the time required for the same amount of elongation, and it indicates a similarity in gradients in respective dissociative state potentials.

The difference in “dissociation time”, we find, is instead due to differences in excited-state repopulation. As shown previously, the initially populated S_6_^1^MLCT state of Fe(CO)_5_ experiences in-phase symmetric stretching leading to periodic population transfer to the lower-energy dissociative ^1^MC states.^7^ We reproduce those results (Figure 3a) and extend the analysis to Cr(CO)_6_ for all singly dissociating trajectories (Figure 3b). In the present study, we simulate the excited-state dynamics of Cr(CO)_6_ resulting from the initially populated S_7_ and S_8_^1^MLCT states. These adiabatic states are two of the electronic states corresponding to the optically bright ^1^T_1u_ transition in the UV spectrum of Cr(CO)_6_. For further details regarding the UV spectra of Fe(CO)_5_ and Cr(CO)6, see Tables S1 and S2. We find that periodic oscillations are present in Cr(CO)_6_ too, but we note the diminished number of trajectories corresponding to contracting Cr–C bonds (compare trajectories at 100 fs). At 100 fs there are 28 trajectories out of 84 which experience a Cr–C contraction. This contrasts with the results of Crespo-Otero et al.^12^ who find only 1 trajectory out of 30 experiencing an excited-state Cr–C contraction. We note, however, that the previous study begins in the lower S_1_–S_3_ states, in contrast to our dynamics starting in the S_7_ and S_8_ excited states. Clearly, in Fe(CO)_5_, we observe a larger portion of the simulated ensemble taking part in oscillations over a longer period of time (up to three oscillations can be discerned as in our earlier report, ref (7)). In Cr(CO)_6_ a smaller portion of the ensemble takes part in oscillations, and the oscillations persist over a shorter time (a maximum of two oscillations can be seen).

We find the Fe–C and Cr–C oscillations to be reflective of the lifetimes of the bound and dissociative states in the adiabatic state populations shown in Figure 3c,d. We color the states there according to bound (blue) or dissociative (red) and group them according to their degeneracies along rigid Fe–C and Cr–C scans (see Figure S1). In Fe(CO)_5_, S_1_–S_2_ and S_3_–S_4_ are degenerate dissociative states, while in Cr(CO)_6_, only S_1_–S_2_ are degenerate dissociative states, with a third nondegenerate S_3_ dissociative state. For Fe(CO)_5_, the initially populated S_6_ state experiences rapid internal conversion to the lower states, as evidenced by the rise of S_3_ and S_4_, but also to higher states, which are bound. This is shown in Figure 3c by two plateaus in the population curves of Fe(CO)_5_ at 45 and 100 fs corresponding to the outer and inner turns of the bound Fe–C oscillations. Additionally, the nondissociative S_5_ state is only transiently populated during the Fe–C oscillations, and the outer turns of the Fe–C stretching cause population to be depleted and subsequently repopulated by the upper nondissociative states. We note that S_1_ and S_2_ are only populated at later times in Fe(CO)_5_, meaning that the first instance of dissociation is largely occurring on S_3_ and S_4_. To illustrate this further, we decomposed the data shown in Figure 3a into the state specific groupings in Figures S2 and S3 to reflect on the adiabatic populations. We find that most trajectories dissociate on S_3_ and S_4_ in Fe(CO)_5_, following internal conversion during the inner turn of the Fe–C oscillation. Additionally, we find with Figure S2 that S_3_ and S_4_ have a fraction which dissociates during the first outer turn and are bound during the inner turn of the Fe–C oscillation. This accounts for the long-lived S_3_ and S_4_ adiabatic population. In the Cr(CO)_6_ adiabatic populations in Figure 3d we note the rapid decay of the initially populated S_7_ and S_8_ states to lower states. While the S_4_–S_6_ nondissociative states and the S_3_ dissociative state are populated, they are only transiently populated as shown by the sharp rise in S_1_ and S_2_. In the state-specific scatter plots for Cr(CO)_6_ in Figure S3, this is seen clearly, where S_3_ is only accounting for a small amount of dissociation, with the bulk of the dissociation occurring on S_1_ and S_2_.

The “later dissociation” in Fe(CO)_5_ can therefore be explained by the longer time that it takes the ensemble to leave the bound electronic states before escaping to the lower dissociative states. In Cr(CO)_6_, a considerable fraction of the ensemble already dissociates at the first oscillation turn after around 50 fs. This is detailed and explained by analyzing the respective populations of bound and dissociative states as a function of time with Figure 3c,d, where blue dashed lines indicate the “dissociation times” (from Figure 2). In Fe(CO)_5_, the populations in bound states take longer to decay (S_6_–S_9_ have decreased to 50% after 50–60 fs), and accordingly, the populations in dissociative states take longer to rise (S_1_ and S_2_ have reached 50% after 150–160 fs, approximately after the “dissociation time” of 155 fs). By contrast, in Cr(CO)_6_, the populations in bound states decay faster (S_7_–S_11_ have decreased by 50% after only 10–20 fs) and populations in dissociative states rise faster (S_1_ and S_2_ have reached 50% after 70 fs, somewhat faster than the “dissociation time” of 95 fs).

In these simulations, we can observe that in Fe(CO)_5_ and 80 fs after initial excitation (which is the time when the 4–5 eV peak is maximal in experiment), we have a significant population of not-yet dissociated configurations. According to the simulated dynamics shown in Figure 3c, 15% of the ensemble is in S_5_ and 30% is in S_6_–S_9_, and hence, 45% of the ensemble has not yet dissociated. As indicated in Figure 3a, most of the Fe–C distances are around 1.8–2 Å at that time. In comparison, in Cr(CO)_6_ and after 80 fs most complexes are dissociated (according to Figure 3d 70% have dissociated; see the 15% in S_3_ and the 55% in S_1_–S_2_, both at 80 fs). As in Fe(CO)_5_, we have significant populations of not-yet dissociated configurations in Cr(CO)_6_ but at significantly shorter times. For comparison, a fraction of around 45% not-yet dissociated configurations is left in Cr(CO)_6_ after 40–50 fs only (Figure 3d with fractions of 30% in S_4_–S_6_ and 15% in S_7_–S_11_, and with, as Figure 3b indicates, a majority of Cr–C distances of around 1.9–2.1 Å). Based on this analysis, we conclude that the 4–5 eV peak seen in experiment in Fe(CO)_5_ at 80 fs after excitation (Figure 1) reflects populations of bound states S_5_–S_9_ with Fe–C distances of around 1.8–2 Å. Due to the faster decay of the bound excited states and because of our limited temporal resolution, we have no clear experimental evidence for the theoretically predicted populations of bound excited states in Cr(CO)_6_ at shorter delays.

Additionally, we think that the observed difference in “dissociation times” is directly related to the couplings of the potential energy surfaces. In our Fe–C and Cr–C rigid scans in Figure S1, the relative changes in the gradients of the bound and dissociative states are negligible, and instead we find that all states for Cr(CO)_6_ are significantly closer in energy. We expand on this further by considering average potential energy surfaces corresponding to the Fe–C and Cr–C distances for all singly dissociating trajectories in Figure S4. It is clear from Figure S4 that, despite perturbations from other degrees of freedom that would widen or narrow the relative energy gaps of the states, all potential energy surfaces are much closer in energy for Cr(CO)_6_ than for Fe(CO)_5_. This is indicative of a higher degree of coupling between the surfaces, leading to an increase in the probability of the individual trajectories to hop between the states. This accounts for the rapid internal conversion between S_7_ and S_8_ to S_1_ and S_2_ in Cr(CO)_6_ relative to the longer-lived dynamics of Fe(CO)_5_.

To better relate the excited-state populations to the measured spectra, we analyze the photoelectron spectra of ground and excited states of Fe(CO)_5_ and Cr(CO)_6_ calculated with CASPT2 for increasing Fe/Cr–C distances in Figure 4 (the species at the largest shown distances of 2.41 Å for Fe(CO)_5_ and 2.50 Å for Cr(CO)_6_ can be regarded as dissociated). In Figure 4, we can observe that the ionization energies are underestimated relative to the measured transient features. We assign this shortcoming of the calculations to limited active spaces and inherent under-estimation of the energy of open-shell systems in CASPT2.^35^

**Figure 4:**
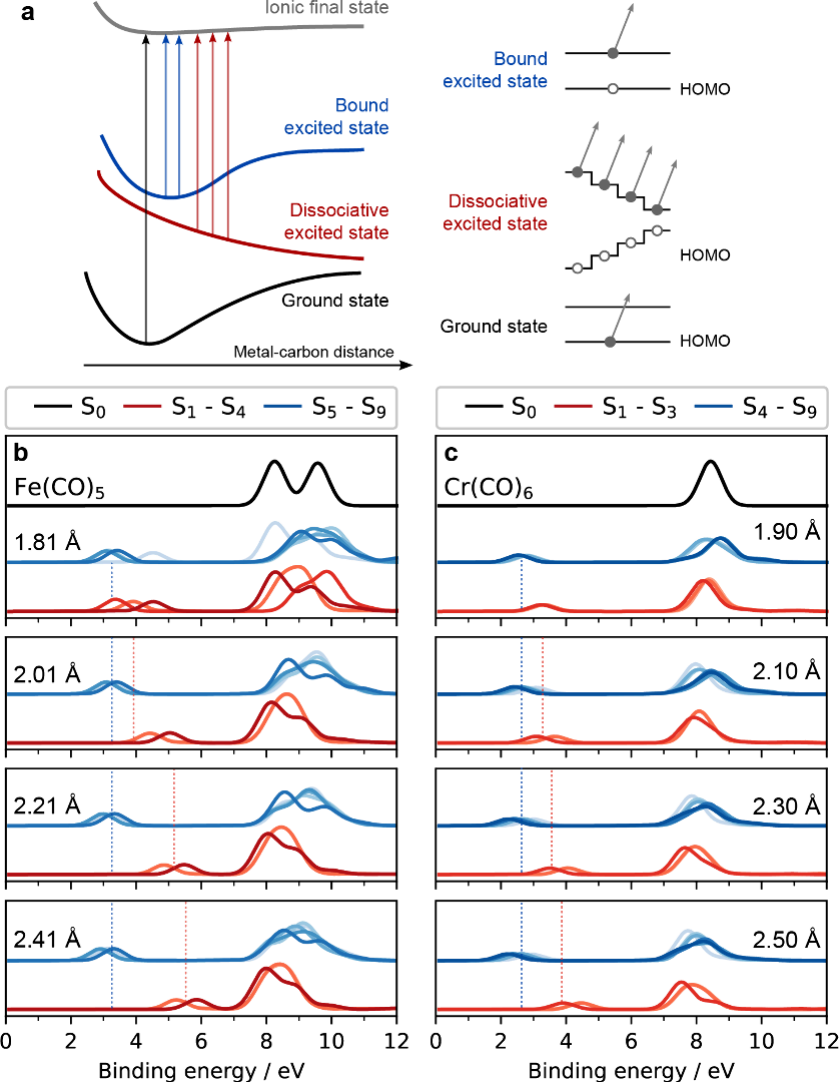
Schematics of the main photoionization processes and calculated photoelectron spectra of the ground and excited states of Fe(CO)_5_ and Cr(CO)_6_. (a) Left: Schematic depiction of ground state, bound and dissociative excited-state and ionized-state potentials as a function of metal–carbon distance. Right: Simplified molecular-orbital energies and electron populations of ground and excited states (bound and dissociative) of Fe(CO)_5_ and Cr(CO)_6_ (HOMO and one higher-lying orbital; arrows indicate photoionization of the “active electron” in the highest-lying occupied molecular orbital). (b and c) Calculated valence photoelectron spectra of ground states (black), bound excited states (blue), and dissociative excited states (red) of Fe(CO)_5_ and Cr(CO)_6_ for the indicated metal–carbon distances, plotted up to the 3d peaks of the ground-state species (vertical lines indicate average binding energies of peaks arising from photoionization of the “active electron” in the highest-lying occupied orbital for bound, blue, and dissociative states, red; integrated intensities in the binding energy range shown are normalized to the same intensities for each metal–C distance; normalization to the ground-state spectra yields indistinguishable results, see Supporting Information Figure S7; for an expanded legend describing the color scheme used for each molecule, see Supporting Information Figure S8).

We reproduce the Fe/Cr 3d peaks of S_0_ ground states of Fe(CO)_5_ and Cr(CO)_6_ with respective equilibrium Fe/Cr–C distances of 1.81/1.9 Å (black spectra in Figure 4b,c). For excited states, we select elongated Fe–C and Cr–C distances from a rigid scan to show how the calculated spectra change. For all excited states and in both species, we see manifolds of peaks in the vicinity of the binding energies of the ground state 3d peaks (8–10 eV in Fe(CO)_5_, 7–8 eV in Cr(CO)_6_). These arise from photoionization from the same 3d orbitals (partially occupied HOMO, HOMO–1, ...) as in the ground states. They appear at similar binding energies as the ground-state 3d peaks because, compared to the ground states and for each system separately, the shift in valence excited-state energies (initial states in the photoionization process) is similar to the shift in ionized-state energies (final states in the process). We do not analyze these further because in the experiment these regions are obscured by background intensities from both ground-state and dissociated species.

Instead, we analyze in detail the new prepeaks in the binding-energy region at 3–7 eV characteristic of excited states arising from photoionization of the “active electron” (here, this is the photoexcited electron in the highest-lying partially occupied orbital in the excited states of Fe(CO)_5_ and Cr(CO)_6_). In the photoionization probing step, we go from the ground-state potential to a continuum of states above the ionization potential and measure the kinetic energies with respect to a given ionic final state. We hence effectively measure the energetic difference between the initial ground- or excited-state potential and the ionic final-state potential. This difference, indicated by vertical arrows in the left side of Figure 4a, equals the measured binding energy. With this simplified potential energy diagram, we deduce that the prepeaks from photoionization of the “active” electron can be interpreted to arise from reaching the same ionic final-state potential albeit starting from different initial-state potentials (for simplicity the ionic final-state potential is assumed to be comparably flat over the entire reaction coordinate given here by the Fe/Cr–C distances). In agreement with our schematic depiction, we see two types of prepeaks from excited states of Fe(CO)_5_ and Cr(CO)_6_ in the calculated photoelectron spectra in Figure 4b,c. First, in the blue spectra of bound excited states, there are peaks around 3 eV in Fe(CO)_5_ (around 2 eV in Cr(CO)_6_) that are constant in binding energy with increasing Fe/Cr–C distance. Second, in the red spectra of dissociative excited states, peaks shift with increasing Fe/Cr–C distance (in Fe(CO)_5_ they shift from around 4 eV at 1.81 Å in the Franck–Condon region to 5 eV at 2.41 Å and in Cr(CO)_6_ from around 2.8 eV at 1.9 Å in the Franck–Condon region to 3.3 eV at 2.5 Å).

At the most elongated distances, we can clearly distinguish between the characters of the adiabatic states (as shown in the rigid scans in Figure 4). For Fe(CO)_5_, at an Fe–C distance of 2.41 Å, we find that the lowest-binding energy peaks of the upper excited ^1^MLCT (bound) states are unperturbed in energy, whereas the lowest-binding energy peaks of the lower dissociative ^1^MC (dissociative) states shift in energy. This opposite behavior can be understood with the scheme in Figure 4a (left), from which it is apparent that in bound excited states of ^1^MLCT character, the changes in potential energy differences (the measured binding energies) are small or negligible when the Fe/Cr–C distances increase or change. The scheme also explains that the peaks from dissociative excited (^1^MC) states are at higher binding energies already in the Franck–Condon region because they arise from photoionization of the relaxed lower-energy states. In addition, the scheme explains that the peaks from dissociative excited states shift to higher binding energies with elongating Fe/Cr–C distances because as the systems proceed on dissociative potential energy surfaces, the difference between initial and final states (the binding energy) progressively increases. For infinite distances the “active electron peak” merges with the peaks of the dissociated species. The measured blue-shift of the 4–5 eV peak in Fe(CO)_5_ at a delay time of 80 fs (after it has reached its maximum intensity) is hence attributed to a progressive dissociation via states S_1_–S_4_ with Fe–C distances from around 2.0 Å to above 2.5 Å. In Cr(CO)_6_ the measured changes are dominated by shifting energies upon dissociation, and the immediate (within our temporal resolution) formation of Cr(CO)_5_ explains the lack of a clear excited-state peak from photoionization of long-lasting bound excited states.

The opposite behaviors of bound and dissociative excited-state peaks and their relation to ground-state peaks can also be understood with an approximate one-electron Koopman’s picture shown in Figure 4a (right).^11^ As drawn, the peaks due to photoionization of the “active” electron in electronic excited states reflect the energies of the highest partially occupied orbital. For both bound and dissociative states, the energy of that orbital is higher than the HOMO energy exclusively occupied in electronic ground states. In the Franck–Condon region, the excited-state peaks hence appear at binding energies lower than those of the ground-state peaks. For bound excited states, the energy of that highest, partially occupied orbital does not change considerably with changes in the Fe/Cr–C distance because covalent interactions do not change considerably. For dissociative excited states, in contrast, the energy of the highest, partially occupied orbital decreases as the overall Fe–Cr–C covalent interactions decrease with increasing Fe/Cr–C distances. The measured binding energy of the “active” electron in that orbital accordingly increases.

In conclusion, our femtosecond valence photoelectron spectra of gas-phase Fe(CO)_5_ and Cr(CO)_6_ exhibit intensities blue-shifting with time after the population of excited states and upon dissociation as initiated by UV excitation. In combination with our excited-state molecular-dynamics simulations and calculated ground and excited-state photoelectron spectra, we find that this is due to populations progressively shifting from bound to dissociative excited states and due to dissociative excited-state energies decreasing as the complexes dissociate. Progression to dissociative states is found to be slower in Fe(CO)_5_ compared to Cr(CO)_6_, explaining the observed “slower dynamics” and, in particular, the longer “dissociation time” in Fe(CO)_5_. In a first attempt to explain this, we argue that the narrow band of dissociative states for Cr(CO)_6_ tightly overlapping with the manifold of bound states enhances coupling and facilitates conversion to dissociative states. In addition, we speculate that differences in angular motions might take the systems away from the structural region of large couplings between bound and dissociative states in different ways, thereby contributing to the observed difference in “dissociation times”. There is a larger flexibility in Fe(CO)_5_ with D_3h_ symmetry compared to the steric hindrance in Cr(CO)_6_ with O_h_ symmetry. The more compact structure in Cr(CO)_6_ would hence not leave room for angular distortion. This hypothesis could be investigated in future high-level calculations of nonadiabatic couplings. Future experiments with better temporal resolution are planned to more directly probe the detailed underlying excited-state and associated non-adiabatic dynamics predicted by our calculations. Such experiments or those employing time-resolved Fe M-edge absorption spectroscopy^36^ have the potential to test the predicted coherent oscillations and bursts of CO release in the dissociation of Fe(CO)_5_ and Cr(CO)_6_, to study the importance of triplet states on longer time scales,^36^ and to determine the mechanisms behind the difference in “dissociation times”.

## Supporting Information Available

Additional data and experimental and computational details (PDF)
Transparent Peer Review report available (PDF)
