# Supplementary material for: Different Photodissociation Mechanisms in Fe(CO)5 and Cr(CO)6 Evidenced with Femtosecond Valence Photoelectron Spectroscopy and Excited-State Molecular Dynamics Simulations
Source: J Phys Chem Lett. 2024 Nov 20;15(47):11830–8. doi: 10.1021/acs.jpclett.4c02025 (PMC11613650; doi:10.1021/acs.jpclett.4c02025)
Supplement: Supplementary file 1 — jz4c02025_si_001.pdf [file jz4c02025_si_001.pdf]

Supplementary Information for:

Different photo-dissociation mechanisms in $Fe(CO)_5$ and $Cr(CO)_6$ evidenced with femtosecond valence photoelectron spectroscopy and excited-state molecular dynamics simulations

Henning Schröder,[†,‡,¶] Michael R. Coates,[§,¶] Raphael M. Jay,[†,‡] Ambar Banerjee,[§,∥] Nomi Sorgenfrei,[‡] Christian Weniger,[‡] Rolf Mitzner,[‡] Alexander Föhlisch,[‡,†] Michael Odelius,[§] and Philippe Wernet[∥]

†Institut für Physik und Astronomie, Universität Potsdam, Haus 28 Karl-Liebknecht-Straße 24/2514476, Potsdam-Golm, Germany

‡Helmholtz-Zentrum Berlin für Materialien und Energie, Hahn-Meitner-Platz 1, 14109 Berlin, Germany

§Department of Physics, Stockholm University, AlbaNova University Center, SE-106 91 Stockholm Sweden

∥Department of Physics and Astronomy, Uppsala University, Box 516, SE-751 20 Uppsala, Sweden

¶*These authors contributed equally to this work.*

E-mail: michael.coates@fysik.su.se; odelius@fysik.su.se; philippe.wernet@physics.uu.se

Results

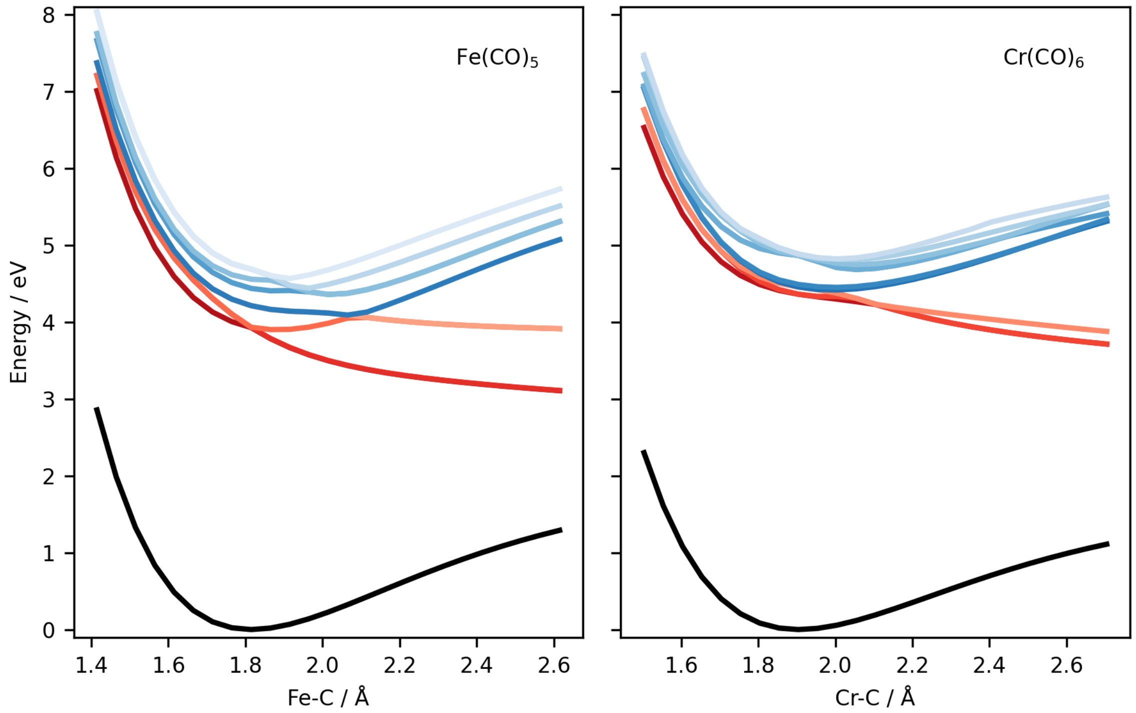

Figure S1: Rigid scan of one of the axial Fe-C distances (left) and one of the Cr-C distances (right) based on structures optimized at the CAM-B3LYP/def2-TZVP level of theory (which was employed in the ESMD simulations). The rigid scans were performed along Fe-C distances of 1.41-2.61 Å and along Cr-C distances of 1.50-2.70 Å. The ground state surfaces were calculated using DFT at the CAM-B3LYP/def2-TZVP level of theory and the excited state surfaces ($Fe(CO)_5$ = S_1-S_9 and $Cr(CO)_6$ = S_1-S_{11}) for both complexes using TDDFT at the same level of theory. For $Fe(CO)_5$, the dissociative S_1-S_4 states are colored red, while the bound S_5-S_9 states are colored blue. Correspondingly for $Cr(CO)_6$, the S_1-S_3 states are colored red, while the S_4-S_{11} states are colored blue. This follows the same color scheme used in the main text.

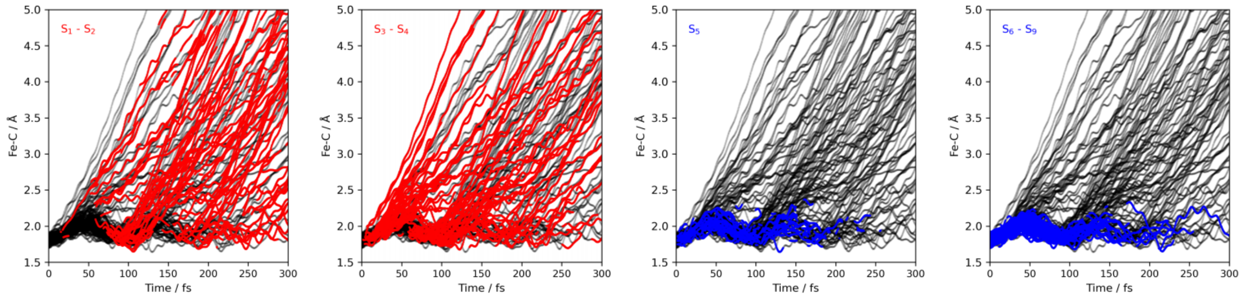

Figure S2: The state specific assignment of the Fe-C distances for all trajectories are plotted on top of the total set of evolving Fe-C distances. The partitioning of the states follows the partitioning defined in Fig. 3(c) for the adiabatic state populations. This follows the same color scheme used in the main text. The data is reproduced from Ref. 1.

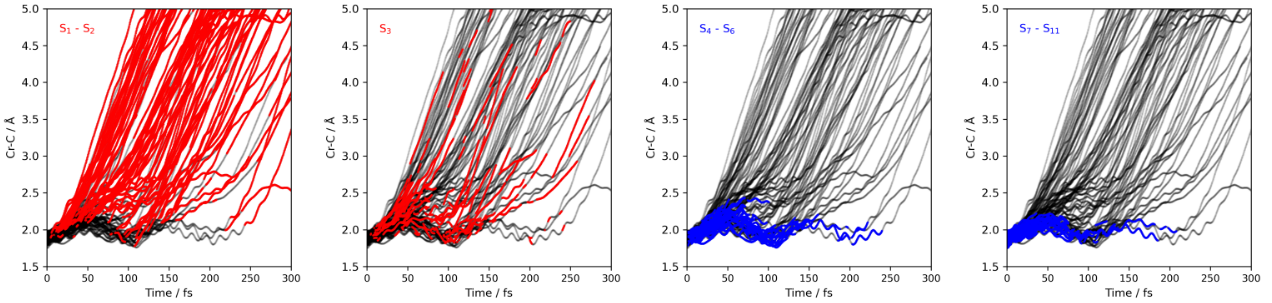

Figure S3: The state specific assignment of the Cr-C distances for all trajectories are plotted on top of the total set of evolving Cr-C distances. The partitioning of the states follows the partitioning defined in Fig. 3(d) for the adiabatic state populations. This follows the same color scheme used in the main text.

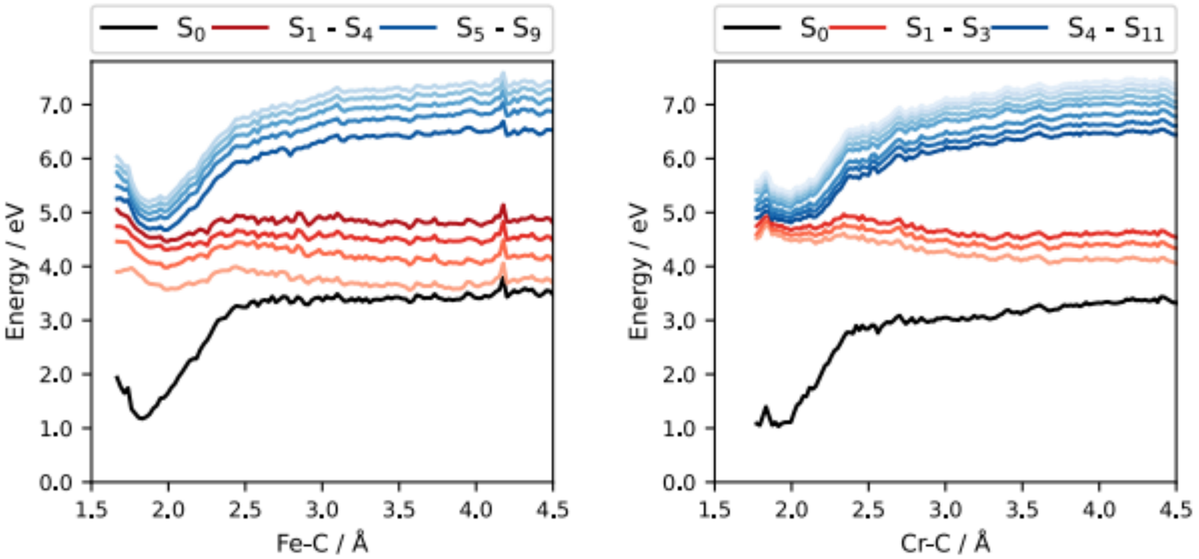

Figure S4: The average potential energy surfaces as based on all dissociating Fe-C (left) and Cr-C (right) distances for all trajectories at all times in the simulation. For $Fe(CO)_5$, the "bound" states are colored blue, corresponding to S_5 - S_9 and the "dissociative" states are colored red, corresponding to S_1-S_4. For $Cr(CO)_6$, the "bound" states are colored blue, corresponding to S_4 -S_{11} and the "dissociative" states are colored red, corresponding to S_1 - S_4. In each case, the reference energy corresponds to the electronic ground state at the reference geometry underlying the Wigner sampling. Data for ironpentacarbonyl is reproduced from Ref. 1.

Experimental details

Photoionization cross sections

In our earlier studies, we probed $Fe(CO)_5$ by photoionization with pulses from the FLASH free-electron laser at a photon energy of 123 eV.[2] Here, we probe at a photon energy of 23 eV. As we show with Fig. S5, at that photon energy, the cross sections for C 2p and O 2p photoionization are much larger than Fe 3d photoionization (the situation is similar for Cr). The correspondingly large intensities from CO valence ionization from both unpumped and pumped $Fe(CO)_5$ or $Cr(CO)_6$ complexes hence prevents us here from analyzing binding-energy regions beyond the low-lying 3d photoelectron peaks.

Calibration of time zero and determination of the temporal resolution

The time zero in the pump-probe experiments was determined by observing side bands in the time-resolved photoelectron spectra of Ar gas measured under the same conditions as $Fe(CO)_5$ and $Cr(CO)_6$ with 266 nm pump and 23 eV probe pulses. The observed side-band intensities, centered around the thereby defined 0 fs time delay, were fitted with Gaussian line profiles. Accuracy of time zero in these fits was ±26 fs. The full-width half-maximum (FWHM) of this Gaussian fit was determined to 260 ± 30 fs and represents the temporal resolution in our experiments.

Calibration of binding energies and spectral resolution

The times of flight in our magnetic-bottle electron spectrometer was converted to calibrated kinetic energies with photoelectron spectra of Ar gas measured in zeroth order of our monochromator spanning high-order harmonics 13 to 21 and Ar 3p photoelectron spectra spanning an according kinetic-energy range (retardation voltage was 3 eV, fundamental photon energy of the 800 nm driver laser was assumed to be 1.55 eV, calibrated binding energies of Ar 3p electrons were taken from literature). Xe 5p photoelectron spectra measured with the 23 eV high-order harmonic were used to determine the overall spectral resolution of the experiments to 400 meV (FWHM of Gaussian line profiles fitted to the Xe 5p photoelectron spectra, representing the combined broadening by the monochromator and the magnetic-bottle electron spectrometer).

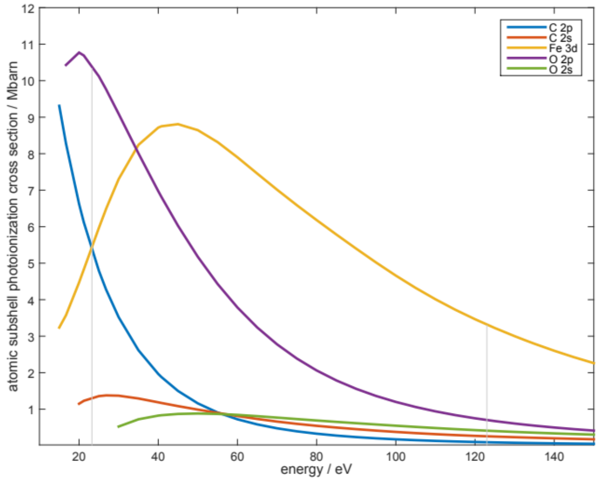

Figure S5: Photoionization cross sections from tabulated values.[3]

UV excitation

The employed pulse energies for the 266 nm pump pulses amounted to 0.5 μJ/pulse with a spot size (round spot) on the sample of 100 μm in diameter (FWHM). This corresponds to a pump fluence of 6.4 mJ/cm^2. This is the same fluence we had used in our previous investigation on $Fe(CO)_5$ in ref. 11 of the main text. As shown there, this is well within the linear regime where pump-probe signals vary linearly with varying pump pulse energy (see Figure 3 in ref. 11 in the main text). And, as described ref. 11 of the main text, this fluence corresponds to an estimated excitation fraction of 6%.

Computational details

The details of the quantum chemistry calculations and SHARC simulations are described in Ref. 1 and we restrict the description here to the $Cr(CO)_6$ computational details.

Ground state geometry and Wigner distribution

Gas-phase optimization of ground state $Cr(CO)_6$ was performed using second order complete active space perturbation theory (CASPT2) with a (10e,10o) active space and the TZVP basis set in Openmolcas[4] and using this structure, the ground state vibrational modes of $Cr(CO)_6$ were obtained in MOLPRO on the B3LYP/cc-pVDZ level of theory to mimic the procedure in our previous study of Fe(CO)5.[5, 6] Using the vibrational modes, a Wigner sampling in phase space was performed twice, the first with 20 sampled geometries and the second with 100 sampled geometries. Based on the combined sampling, the UV spectrum was constructed from discrete transitions using TDDFT at the CAM-B3LYP/def2-TZVP level of theory to a total of 9 singlet excited states.

Calculated UV spectral assignment

State	TDDFT (eV)	Osc. Str.	Character
$^1A_1''$ (MLCT)	3.89	0.000	10e′→4e″*
$^1E'$ (MC)	3.97	0.000	10e′→14a$_1$′*
$^1E''$ (MLCT)	4.17	0.000	10e′→4e″*
$^1A_2''$ (MLCT)	4.40	0.067	10e′→4e″*

Table S1: Calculated CAM-B3LYP/def2-TZVP excited state energies of $Fe(CO)_5$ at the CASPT2 optimized D_{3h} geometry.

State	TDDFT (eV)	Osc. Str.	Character
^1E_u (MLCT)	4.34	0.000	$2t_{2g}\rightarrow 9t_{1u}^*$
$^1T_{2u}$ (MLCT)	4.44	0.000	$2t_{2g}\rightarrow 9t_{1u}^*$
$^1A_{2u}$ (MLCT)	4.47	0.000	$2t_{2g}\rightarrow 9t_{1u}^*$
$^1T_{1u}$ (MLCT)	4.84	0.0243	$2t_{2g}\rightarrow 9t_{1u}^*$

Table S2: Calculated CAM-B3LYP/def2-TZVP excited state energies of $Cr(CO)_6$ at the CASPT2 optimized O_h geometry.

The analysis of the transitions of the UV spectra for both molecules are presented in Table S1 and S2. In $Fe(CO)_5$, the manifold of states presented in Table S1 corresponds to 6 adiabatic electronic states S_1 – S_6, which includes the optically bright ^{1}MLCT state in S_6. In Cr(CO)6, the manifold of states presented in Table S2 corresponds to 9 adiabatic electronic states S_1 – S_9, which includes the optically bright S_7, S_8, and S_9 ^{1}MLCT states.

SHARC simulations

The $Cr(CO)_6$ ESMD simulations were performed using the semi-classical surface hopping method implemented in the SHARC version 2.1 code.[7-9] The Wigner sampling and subsequent UV spectrum calculations resulted in 42 trajectories being excited into each of the S_7 and S_8 optically bright ^{1}MLCT states, giving a total of 84 trajectories from these two initial states. The 84 trajectories were propagated from the S_7 and S_8 states accessing a total of 10 singlet excited states and the singlet ground state. Each trajectory was propagated for up to 1000 fs using a timestep of 0.5 fs but many trajectories were killed off at earlier timesteps due to convergence failure in the SCF step following large amplitude Cr-C distances associated with excited state dissociation. The remaining details of the SHARC simulations (non-adiabatic couplings, velocity rescaling, hopping probabilities) are identical to those described in Ref. 1.

Quantum Chemistry

The energies and forces guiding each trajectory were based on the CAM-B3LYP/def2-TZVP level of theory with RIJCOSX for computational efficiency. These calculations were performed using ORCA 4.2.0[10] which was interfaced to the SHARC code.[7-9] The calculation of photoelectron spectra for both $Fe(CO)_5$ and $Cr(CO)_6$ were performed on structures optimized at the CAM-B3LYP/def2-TZVP level of theory. Static photoelectron spectra were calculated at the CASPT2 level of theory based on a complete active space self-consistent field method (CASSCF) wavefunction with the ANO-RCC-VDZP basis set. We employed a (10e,10o) active space to be in agreement with the active space used in Ref. 1 and with Pierloot et al.[10] This active space for $Fe(CO)_5$ was applicable also to $Cr(CO)_6$ where the active space composition was taken to include the $5e_g(\sigma_{CO} - d_z2,\ \sigma_{CO} - d_x2{-}y2)$ and $2t_{2g}(d_{xy} - \pi CO^*,\ d_{xz} - \pi CO^*,\ d_{yz}\ - \pi CO^*)$ occupied orbitals and the $3t_{2g}(d_{xy} - \pi CO^*,\ d_{xz} - \pi CO^*,\ d_{yz} - \pi CO^*)$ and $6e_g(d_z2 - \sigma CO^*,\ d_x2{-}y2 - \sigma CO^*)$ unoccupied orbitals. The CASPT2 calculations were performed in Openmolcas v23.02[4] using an IPEA shift of 0.25 and an imaginary shift of 0.2. All simulations were performed without symmetry in the C_1 point group. A state averaging of 10 singlet states was used for all of the neutral calculations and 100 doublet states for the cationic state calculations, followed by perturbation theory calculations. The state energy differences between the manifolds of singlet and doublet states were calculated and relative probabilities of ionization (transition intensities) were taken to be proportional to the square of the Dyson orbital norms.[11] Each spectrum was then subsequently broadened using a pseudo-Voigt function with a Lorentzian contribution of 0.0005 eV half-width half-maximum (HWHM) and a Gaussian contribution of 0.4 eV HWHM. The ground and excited state photoelectron spectra were calculated at the CASPT2 level of theory along a set of geometries obtained from the rigid scans in Fig. S1.

The choice of a CAS(10e,10o) active space for both molecules allows for an even comparison of the potential energy surfaces for the low-lying valence states. We selected this active space based on previous analysis in the supplementary information of Banerjee et al.[1] where the CAM-B3LYP/def2-TZVP level of theory was shown to have a good agreement with the second order n-electron valence state perturbation theory (NEVPT2)[12,13] based on an underlying CAS(10e,10o) wavefunction. With respect to $Cr(CO)_6$, the CASPT2 potential energy surfaces based on an underlying CAS(10e,10o) active space show a comparable agreement to the CAM-B3LYP/def2-TZVP surfaces in Fig. S1. In the construction of the active space, we neglected the set of triply degenerate $9t_{1u}$ orbitals that give rise to the set of initial electronic states formed by excitation in the Franck-Condon region. The active space includes a set of $3d_{z^2}$ and $3d_{x^2-y^2}$ orbitals which are necessary in the description of the dissociation of the Fe-CO bond. Nevertheless, the potential energy surfaces for $Cr(CO)_6$

provide the correct set of three dissociative states (red) and the remaining set of non-dissociative states (blue) as shown in Fig. S6.

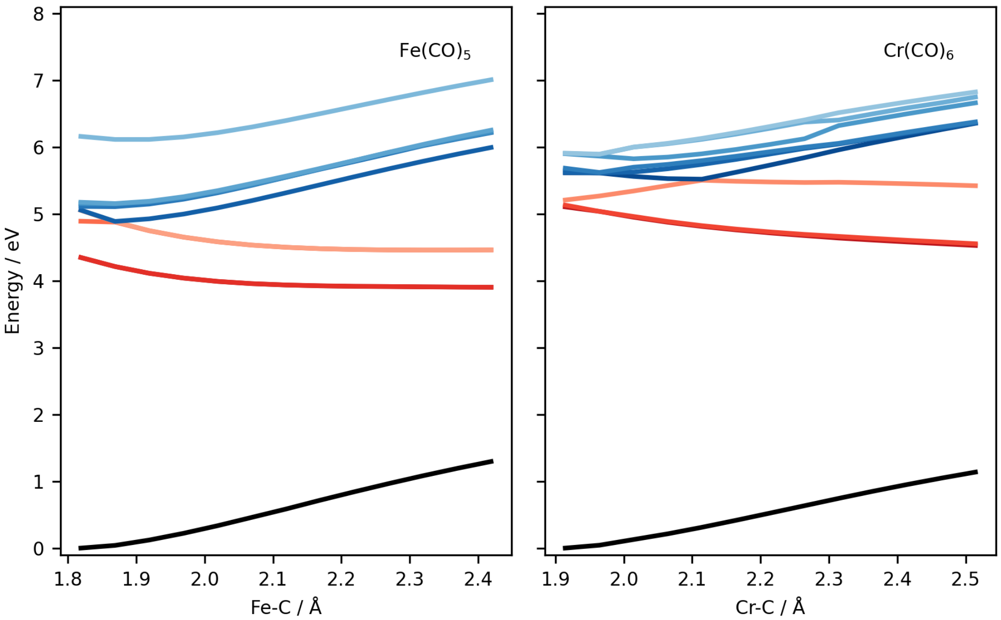

Figure S6: Rigid scan of one of the axial Fe-C distances (left) and one of the Cr-C distances (right) based on structures optimized at the CAM-B3LYP/def2-TZVP level of theory (which was employed in the ESMD simulations). The rigid scans were performed along Fe-C distances of 1.81-2.41 Å and along Cr-C distances of 1.91-2.51 Å. The ground state surfaces were calculated using at the CASPT2 level of theory based on an underlying CAS(10e,10o) wavefunction and with a ANO-RCC-VDZP basis set. The excited state surfaces (S_1-S_9) were obtained along with the ground state via a state-averaged wavefunction averaged over 10 states in total. For $Fe(CO)_5$, the dissociative S_1-S_4 states are colored red, while the bound S_4-S_9 states are colored blue. Correspondingly for $Cr(CO)_6$, the S_1-S_3 states are colored red, while the S_4-S_9 states are colored blue. This follows the same color scheme used in the main text.

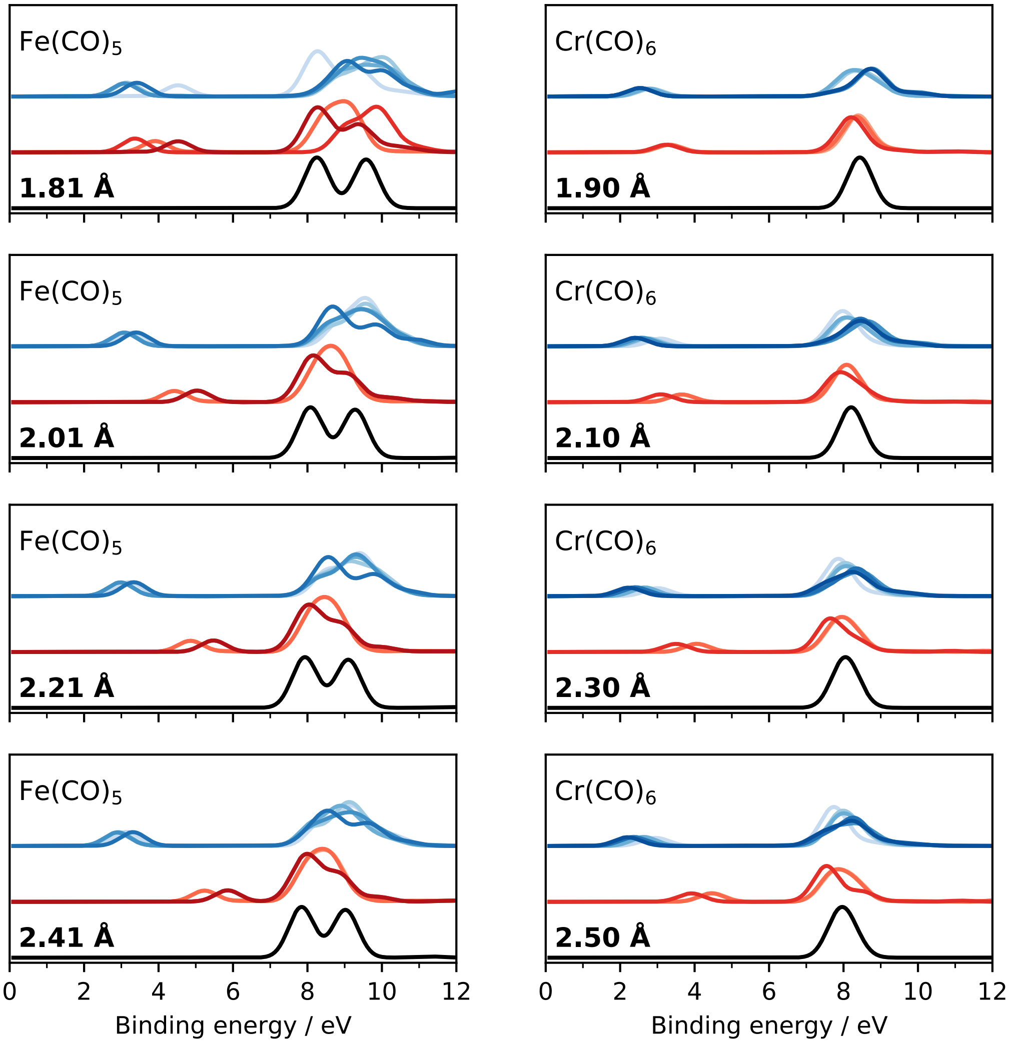

Figure S7: Calculated valence photoelectron spectra of ground states (black), bound excited states (blue) and dissociative excited states (red) of $Fe(CO)_5$ and $Cr(CO)_6$ for the indicated metal-carbon distances, plotted up to the 3d peaks of the ground state species. The integrated intensities in the range shown are normalized to the same intensities as the ground state photoelectron spectrum at the Franck-Condon geometry ($Fe(CO)_5$: Fe-C = 1.81 Å and $Cr(CO)_6$: Cr-C = 1.90 Å).

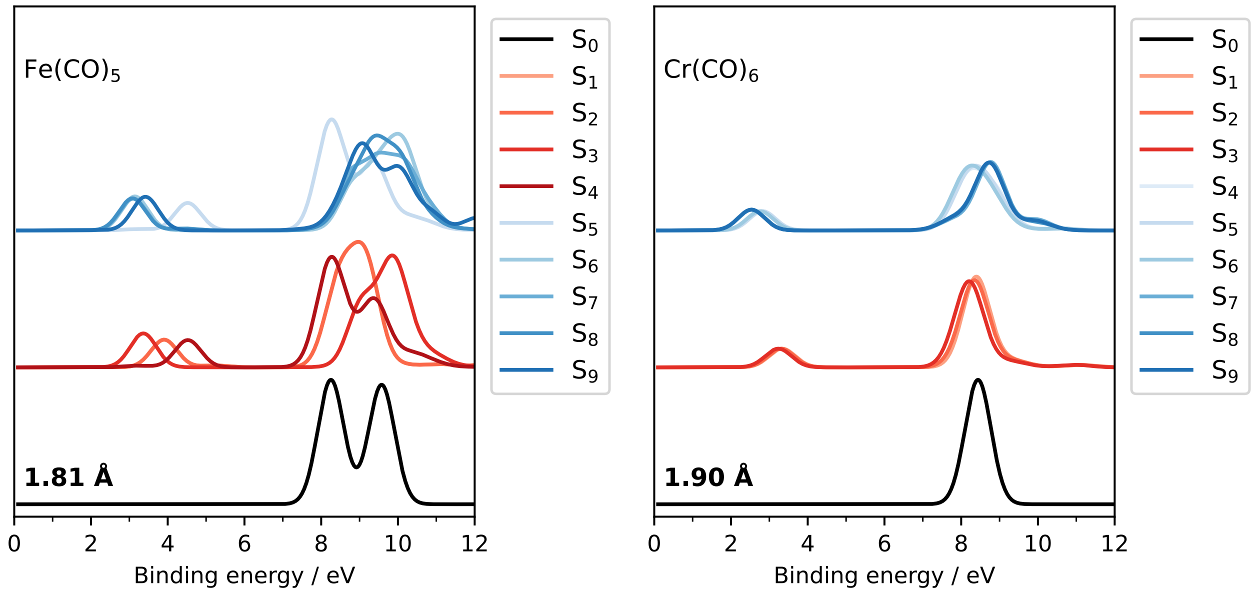

Figure S8: Calculated valence photoelectron spectra of ground states (black), bound excited states (blue) and dissociative excited states (red) of $Fe(CO)_5$ and $Cr(CO)_6$ for the Franck-Condon geometries ($Fe(CO)_5$: Fe-C = 1.81 Å and $Cr(CO)_6$: Cr-C = 1.90 Å) of each molecule plotted up to the 3d peaks of the ground state species. The integrated intensities in the range shown are normalized to the same intensities as the ground state photoelectron spectrum. The expanded legend is included to indicate the color scheme used in Figure 4 in the main text of the paper.

References

(1) Banerjee, A.; Coates, M. R.; Kowalewski, M.; Wikmark, H.; Jay, R. M.; Wernet, P.; Odelius, M. Photoinduced bond oscillations in ironpentacarbonyl give delayed synchronous bursts of carbonmonoxide release. *Nat. Commun.* **2022**, *13*, 1337.

(2) Leitner, T.; Josefsson, I.; Mazza, T.; Miedema, P. S.; Schröder, H.; Beye, M.; Kunnus, K.; Schreck, S.; Düsterer, S.; Föhlisch, A.; Meyer, M.; Odelius, M.; Wernet, P. Time-resolved electron spectroscopy for chemical analysis of photodissociation: Photoelectron spectra of $Fe(CO)_5$, $Fe(CO)_4$, and $Fe(CO)_3$. *J. Chem. Phys.* **2018**, *149*, 044307.

(3) Yeh, J.; Lindau, I. Atomic subshell photoionization cross sections and asymmetry parameters: $1 \leqslant Z \leqslant 103$. *At. Data Nucl. Data Tables* **1985**, *32*, 1-155.

(4) Li Manni, G. et al. The OpenMolcas Web: A Community-Driven Approach to Advancing Computational Chemistry. *J. Chem. Theory Comput.* **2023**, *19*, 6933-6991.

(5) Werner, H.-J. et al. The Molpro quantum chemistry package. *J. Chem. Phys.* **2020**, *152*, 144107.

(6) Werner, H.-J.; Knowles, P. J.; Knizia, G.; Manby, F. R.; Schütz, M. Molpro: a general-purpose quantum chemistry program package. *WIREs Comput. Mol. Sci.* **2012**, *2*, 242-253.

(7) Mai, S.; Marquetand, P.; Gonzalez, L. Nonadiabatic dynamics: The SHARC approach. *WIREs Comput. Mol. Sci.* **2018**, *8*, e1370.

(8) Mai, S.; Marquetand, P.; Gonźalez, L. A general method to describe intersystem crossing dynamics in trajectory surface hopping. *Int. J. Quantum Chem.* **2015**, *115*, 1215- 1231.

(9) Richter, M.; Marquetand, P.; Gonźalez-Váquez, J.; Sola, I.; Gonźalez, L. SHARC: Ab Initio Molecular Dynamics with Surface Hopping in the Adiabatic Representation Including Arbitrary Couplings. *J. Chem. Theory Comput.* **2011**, *7*, 1253-1258. Neese, F.; Wennmohs, F.; Becker, U.; Riplinger, C. The ORCA quantum chemistry program package. *J. Chem. Phys.* **2020**, *152*, 224108.

(10) Pierloot, K. The CASPT2 method in inorganic electronic spectroscopy: from ionic transition metal to covalent actinide complexes. *Mol. Phys.* **2003**, *101*, 2083-2094.

(11) Pickup, B. T. On the theory of fast photoionization processes. *Chem. Phys.* **1977**, *19*, 193-208.

(12) Dyall, K. G. The choice of a zeroth-order Hamiltonian for second-order perturbation theory with a complete active space self-consistent-field reference function. *J. Chem. Phys.* **1995**, *102*, 4909-4918

(13) Angeli, C.; Cimiraglia, R.; Evangelisti, S.; Leininger, T.; Malrieu, J.-P. Introduction of n-electron valence states for multireference perturbation theory. *J. Chem. Phys.* **2001**, *114*, 10252-10264
