# Supplementary material for: Different Photodissociation Mechanisms in Fe(CO)5 and Cr(CO)6 Evidenced with Femtosecond Valence Photoelectron Spectroscopy and Excited-State Molecular Dynamics Simulations
Source: J Phys Chem Lett. 2024 Nov 20;15(47):11830–8. doi: 10.1021/acs.jpclett.4c02025 (PMC11613650; doi:10.1021/acs.jpclett.4c02025)
Supplement: Supplementary file 2 — jz4c02025_si_002.pdf [file jz4c02025_si_002.pdf]

jz-2024-02025d.R1

Name: Peer Review Information for "Different photo-dissociation mechanisms in Fe(CO)5 and Cr(CO)6 evidenced with femtosecond valence photoelectron spectroscopy and excited-state molecular dynamics simulations"

First Round of Reviewer Comments

Reviewer: 1

Comments to the Author

This manuscript describes the photolysis of the title compounds based on experimental valence photoelectron spectroscopy (PES) with 260 fs time resolution and supporting molecular dynamics simulations up to 300 fs. The MD simulations nicely explain the experimental findings in terms of a slower dissociation for the Fe complex due to slower internal conversion from non-dissociative to dissociative electronic states, which is consistent with a blue-shift of the binding energies in the experimental measurements. The combination of experimental and computational results is insightful and provides a nice picture for the interesting ultrafast dynamics of these systems.

Although this is interesting work and is technically sound, it is probably more suitable for a more specialized journal like JPC-A. Both the experiments and the computational work are challenging and cutting edge, but the discussion is somewhat descriptive in places and lacks specific information about the internal conversion processes that would enable electronic relaxation. For example, the character of the states S7, S6, etc are not described in a meaningful level of detail and it's not clear why the states are grouped together in the way they are for Figure 3, except to separate dissociative and bound states. At least some computational details should be provided in the main text to describe how the state couplings are handled. Moreover, the simplified energy level diagram in Figure 4 seems to contradict the multistate description of the dynamics involving as many as 8-10 electronic states (eg, in Figure 3).

The schematic diagram in Figure 4 is helpful to illustrate the underlying explanation of the experimental PES (namely the different behaviors for bound and dissociated states), but it's unclear how to explain the lack of very low binding energy bands in the PES. The calcualtions show as low as 2 eV, but the authors go out of their way to explain that there are no experimental bands observed below ~4 eV. Maybe I'm missing something here, but there seems to be a disconnect on this point. In other words, it is rather unsatisfying to say that the experiment only probes the lower lying electronic states S1-S4, when much of the dynamics occurs starting much higher.

On page 9, lines 9-17 - The description of a “time-independent” band due to “spillover” intensity is very confusing. The band that appears at this energy range 5-6 eV clearly does change in time. I think the authors are only referring to an underlying contribution, but this comment comes on the heels of a discussion about “side-bands” that would only appear at t=0, so it is not clear what the authors actually mean.

Also on page 9, line 28 - suggesting that there is a “fundamentally different photo-dissociation mechanism” is an overstatement. The difference is more about time scales than mechanism. In both systems the photoelectron band blueshifts during electronic relaxation and then decays due to photolysis. There is nothing fundamentally different between these, even if the observables tend up looking a little different.

In Figure 3, what is meant by “fraction of survival”? Is this the fraction of population remaining, or the number of trajectories that didn’t fail?

Symmetry arguments in the conclusion are interesting, but would be more impactful if they were supported with some evidence from the calculations or otherwise. The authors say that this could be a good target for higher level calculations, but the result must somehow be evident from the current calculations because they already predict the different lifetimes!

In short, this is good work and an interesting contribution. A few minor details should be addressed before publication in a more specialized journal, such as JPC-A.

Reviewer: 2

Comments to the Author

The paper by Schroeder and colleagues provides a thorough comparative analysis of the ultraviolet-induced dissociation of two well-known ultrafast model systems, FeCO5 and CrCO6. The study is organized into two parts. The experimental section, though concise, presents time-resolved valence photoelectron spectra for both molecules. There are notable differences between the spectra of FeCO5 and CrCO6, particularly in terms of the minimum binding energy and the time constants for cuts at various binding energies. Even though the time resolution of 260 fs is relatively long for this type of experiment—much longer than in some previous studies—a clear distinction is still evident. Specifically, CrCO6 shows less intensity accumulation at low binding energies compared to FeCO5, and the decay rates are faster in CrCO6.

The experimental work is complemented by a detailed simulation study that explores the dissociation dynamics of the two molecules. The simulations suggest that dissociation occurs in bursts, driven by vibrational wave packet motion. They also identify the electronic states involved in the dissociation process, distinguishing between bound and dissociative states. The authors propose that the transitions from the initially excited bound states to dissociative states are the rate-limiting step for dissociation. The faster dissociation observed in CrCO6 compared to FeCO5 is attributed to the denser electronic states in CrCO6, which facilitate these transitions.

Overall, the paper is well-written, and the message is clear. However, the time resolution of 260 fs is not sufficient to fully resolve the dynamics experimentally, and it is much longer than the current state in similar experiments with the HHG sources. The trajectory simulations in Fig. 3 indicate that a resolution closer to 50 fs would be more appropriate, and it could in principle be reached. The theoretical discussion is somewhat disconnected from the experimental results, likely due to this limitation. While the authors may not be able to address this issue, I would still recommend publishing the paper in this journal, as the study of these prototype molecules is valuable.

I have a few additional, more minor comments:

1. The lack of signal in the binding energy range of 5.5-6 eV for FeCO5 is not sufficiently discussed. This is a prominent feature that should be addressed.

2. What was the excitation fluence, and what is the expected molecular excitation density? Did the authors conduct a UV power titration?

3. I suggest using the terms “shift to higher/lower binding energies” instead of “red” or “blue.”

4. On page 9, last paragraph: How can the maximum be at -60 fs when the time zero was previously calibrated with another sample?

5. The authors should justify the comparison of delay traces at the same binding energy, as mentioned at the bottom of page 11. The vertical IP differs by only 0.2 eV, which justifies the comparison. However, if the IP differed by one bin size, the dynamics in adjacent bins should be compared.

6. It would be helpful if the authors discussed the optically allowed transitions to states S1-S11. Clearly, the transitions to the red-dissociative states are not optically allowed.

7. Figure 4 would benefit from a legend explaining the different colors.

Author's Response to Peer Review Comments:

The Journal of Physical Chemistry
Letters
Senior Editor

Prof. Philippe Wernet

Uppsala University
Department of Physics and Astronomy
Chemical and Bio-Molecular Physics

Box 516
751 20 Uppsala
Sweden

Visiting address:
Ångströmlaboratoriet Lägerhyddsvägen 1

Phone:
+46 72 9999707

www.physics.uu.se

philippe.wernet@physics.uu.se

SUBMISSION OF OUR REVISED MANUSCRIPT ID jz-202402025d

17 October 2024

Dear Editor,

We thank you very much for your interest in our work and we thank the reviewers for their constructive feedback. That was very helpful for us in improving our manuscript. We are sending you here a revised version of this manuscript (ID jz-2024-02025d) together with a point-by-point answer to the points raised by the reviewers.

With this we hope that you find our manuscript suitable for publications in the *Journal of Physics Chemistry Letters* and we are looking forward to your assessment.

Sincerely yours,

Philippe Wernet (on behalf of all authors)

Revision letter for manuscript ID jz-2024-02025d

We are grateful for the careful reading by the editor and reviewers and have a revised version of the manuscript. We confirm here that we have addressed all of the reviewer comments by presenting revised data analysis, supporting calculations and we have highlighted the changes made in the manuscript. We address below all comments point-by-point for each reviewer.

In addition to the changes suggested by the reviewers, we have made the following small corrections:

- We found that the script used to plot the theoretical photoelectron spectra in the original version of the manuscript contained an error. The ground-state photoelectron spectra of both $Fe(CO)_5$ and $Cr(CO)_6$ were accidentally added to the groups of ^{1}MLCT state spectra (blue) in Figure 4. This has been corrected and we note that this has no impact on the interpretation and discussion of the photoelectron spectra.
- We additionally shifted all calculated photoelectron spectra in Figure 4 to match the experimental ground state spectra of each molecule (shifts of +0.35 eV and +0.55 eV were applied for $Fe(CO)_5$ and $Cr(CO)_6$, respectively). This improves comparison of calculated and experimental spectra and further supports our conclusions.

- We added the new reference 36 (Tross et al.) which we find an important new reference on the gas-phase dynamics of $Fe(CO)_5$ that relates well to our study (see changes in the conclusion).
- We corrected several typos throughout (see tracked changes in the revised manuscript).
- We renumbered the references as we moved one in the list and we added a new reference.

Response to Reviewer #1:

This manuscript describes the photolysis of the title compounds based on experimental valence photoelectron spectroscopy (PES) with 260 fs time resolution and supporting molecular dynamics simulations up to 300 fs. The MD simulations nicely explain the experimental findings in terms of a slower dissociation for the Fe complex due to slower internal conversion from non-dissociative to dissociative electronic states, which is consistent with a blue-shift of the binding energies in the experimental measurements. The combination of experimental and computational results is insightful and provides a nice picture for the interesting ultrafast dynamics of these systems.

Although this is interesting work and is technically sound, it is probably more suitable for a more specialized journal like JPC-A. Both the experiments and the computational work are challenging and cutting edge, but the discussion is somewhat descriptive in places and lacks specific information about the internal conversion processes that would enable electronic relaxation. For example, the character of the states S7, S6, etc are not described in a meaningful level of detail and it's not clear why the states are grouped together in the way they are for Figure 3, except to separate dissociative and bound states. At least some computational details should be provided in the main text to describe how the state couplings are handled. Moreover, the simplified energy level diagram in Figure 4 seems to contradict the multistate description of the dynamics involving as many as 8-10 electronic states (eg, in Figure 3).

We appreciate the feedback from the reviewer. In the Supplementary Information, we have added tables describing the character of the valence-excited states at the ground state geometry of Fe(CO)5 and Cr(CO)6. In the construction of the initial conditions for the molecular dynamics simulations, we sample a ground state Wigner distribution to determine the position and momenta. In the vicinity of the Franck-Condon region, the states are well defined. The characters are not well defined, however, during the molecular dynamics simulations since these are adiabatic electronic states. We instead refer to the grouping of states like in Figure 3 or in the average potential energy surfaces in Figure S4.

In the excited state molecular dynamics simulations, the wavefunction overlap method is used to follow non-adiabatic transitions. Hence, we have not properly calculated non-adiabatic couplings. (see response to Reviewer 2 for more details.)

With respect to Figure 4, we regret the misunderstanding. We show only two curves as representative cases to illustrate the characteristic differences of the respective states and to avoid confusion by the larger number curves necessary to illustrate the more complicated (and realistic) case probed in experiment with, in addition, crossings of curves in the manifolds of bound and dissociative valence-excited states.

We hope the reviewer agrees that this may be the best we can do at this moment in conceptually describing the states and, at the same time, illustrating the basic spectroscopic principle observed here.

Explicit changes to the manuscript: Table S1 and S2 have been added to the Supporting Information file, including a small summary of the tables. We have additionally referenced this in the main text on page 14.

The schematic diagram in Figure 4 is helpful to illustrate the underlying explanation of the experimental PES (namely the different behaviors for bound and dissociated states), but it's unclear how to explain the lack of very low binding energy bands in the PES. The calcualtions show as low as 2 eV, but the authors go out of their way to explain that there are no experimental bands observed below ~4 eV. Maybe I'm missing something here, but there seems to be a disconnect on this point. In other words, it is rather unsatisfying to say that the experiment only probes the lower lying electronic states S1-S4, when much of the dynamics occurs starting much higher.

We thank the reviewer for pointing this out. We believe that this is the result a combination of effects. First, we noticed that the calculated spectra in Figure 4 of the original version of the manuscript we not correctly shifted in energy to best match the experimental spectra. We now provide a new Figure 4 with the revised version of our manuscript where calculated spectra were shifted to match the calculated and measured ground-state spectra. In this revised figure, the lowest binding energy peaks are now close to 3 eV corresponding to the lowest binding energy for which we see intensity in experiment (see Figure 1). Measured intensities at 3 eV are small, probably because the population of states that contribute there are small at the measured time delays. In addition, short-time dynamics are smeared due to our comparably poor temporal resolution, we think. Future experiments will hopefully reveal more details about the states populated at very early times. Second, our level of theory chosen in the calculation affects the accuracy of calculated binding energies. Our CASPT2 calculations predict too low of a binding energy for all states when compared to the experiment. CASPT2 is known to generally under-estimate the energies of states when going from a closed shell system ($Fe(CO)_5$/$Cr(CO)_6$) to an open shell system

($Fe(CO)_5^+$/$Cr(CO)_6^+$) as described by Ghigo *et al. Chem. Phys. Lett.* **2004**, *396* (1), 142-149 (now reference 35 in our revised manuscript). This is partially improved by the so-called IPEA shift (Zobel *et al. Chem. Sci.* **2017**, *8* (2), 1482-1499.) which we have employed in the current calculations using a

value of 0.25. Nevertheless, we believe the energies of the cationic states are too low in energy. We also believe this is partly due to a limitation of the active space chosen for the underlying CASSCF calculation which is selected to be compatible with both $Fe(CO)_5$ and $Cr(CO)_6$. Hence, we instead choose to focus on the trends of the binding energies which show that the upper ^{1}MLCT states are largely unshifted by the Fe-C/Cr-C dissociation, while the lower ^{1}MC states shift to higher binding energies.

<u>**Explicit changes to the manuscript:**</u> Two sentences added to manuscript on page 18. "In Figure 4, we can observe that the ionization energies are underestimated relative to measured transient features. We assign this short-coming of the calculations to limited active spaces and inherent under-estimation of the energy of open-shell systems in CASPT2 [Ghigo *et al. Chem. Phys. Lett.* **2004**, *396* (1), 142-149]."

On page 9, lines 9-17 - The description of a "time-independent" band due to "spillover" intensity is very confusing. The band that appears at this energy range 5-6 eV clearly does change in time. I think the authors are only referring to an underlying contribution, but this comment comes on the heels of a discussion about "side-bands" that would only appear at t=0, so it is not clear what the authors actually mean.

We thank the reviewer for pointing out how confusing this text is and we changed that section to, hopefully, make it clearer,

<u>**Explicit changes to the manuscript:**</u> The text on page 7 was changed to: "An additional complication in our experiment masks the targeted dynamics. Spectra of $Cr(CO)_6$ at -240 and 500 fs in Fig. 1f show that part of the intensity at 5-6 eV is time-independent (part of the intensity in this region does not change). This is also visible in Fig. 1d as a faint light-blue vertical intensity band underlying the data for all times at 5-6 eV. This time-independent intensity portion originates from photoionization of ground-state $Cr(CO)_6$ by another harmonic than the main selected one at 23 eV due to "spill-over" in our monochromator (light of another photon energy than the nominally selected harmonic ionized $Cr(CO)_6$. These spurious intensities are more apparent in $Cr(CO)_6$ compared to $Fe(CO)_5$ since overall pump-probe intensities are much smaller in Cr compared to Fe)."

Also on page 9, line 28 - suggesting that there is a "fundamentally different photo-dissociation mechanism" is an overstatement. The difference is more about time scales than mechanism. In both systems the photoelectron band blueshifts during electronic relaxation and then decays due to photolysis. There is nothing fundamentally different between these, even if the observables tend up looking a little different.

Yes, we agree with this assessment. We certainly do not want to over-sell our results.

Explicit changes to the manuscript: We changed that sentence on page 8 to "Our data in Fig. 1c and d with detailed views in Fig. 1e and f clearly reflects differences in the photo-dissociation mechanisms of $Fe(CO)_5$ and $Cr(CO)_6$." And we hope the reviewer agrees that differences in populations and lifetimes of excited states and differences in which the systems leave the respective manifolds of states via coupled potential energy curves can be termed differences in mechanisms.

In Figure 3, what is meant by "fraction of survival"? Is this the fraction of population remaining, or the number of trajectories that didn't fail?

We thank the reviewer for asking for clarification as we understand that this might be confusing. The fraction of survival is a display of the number of active trajectories at each time step, since many trajectories are terminated due to SCF convergence failure after the M-CO distances become too large. At each time, the populations are normalized to the number of parent trajectories, not the number of active trajectories. Since the majority of trajectories experience rapid dissociation involving large elongation of the M-CO bonds, the fraction of survival reflects upon the dynamics of intact trajectories at longer times.

Explicit changes to the manuscript: On page 11 in the main text we have now defined the fraction of survival.

Symmetry arguments in the conclusion are interesting, but would be more impactful if they were supported with some evidence from the calculations or otherwise. The authors say that this could be a good target for higher level calculations, but the result must somehow be evident from the current calculations because they already predict the different lifetimes!

We thank the reviewer for pointing this out, but we are afraid that what the reviewer expectations goes well beyond the scope of our investigation and, in fact, beyond what we think is currently feasible. Within the framework of the surface hopping simulations, the couplings between electronic states are calculated using wavefunction overlap methods, rather than the explicit calculation of the nonadiabatic couplings. At the TDDFT level of theory employed in the present study, we cannot calculate the nonadiabatic couplings. We therefore speculate that at a higher level of theory (CASPT2/MRCI), the nonadiabatic couplings would explain the coupling between states. Presently, such a level of theory would not be computationally feasible to simulate dynamics. Furthermore, the selection of a correct active space in an underlying CASSCF calculation would require a much more rigorous study. We employ a smaller active space for

$Cr(CO)_6$ in this study when compared to what is suggested by Ben Amor *et al. Chem. Phys. Lett.* **2006,** *421* (4), 378-382. The authors suggest an active space for CASSCF containing 10 electrons in 17 orbitals. To obtain correct excitation energies of each of the states, a subsequent CASPT2 or MRCI calculation would be necessary, however, an active space of this size would prohibit that calculation. The details of our active space are presently described in the supporting information file.

Response to Reviewer #2:

The paper by Schroeder and colleagues provides a thorough comparative analysis of the ultraviolet-induced dissociation of two well-known ultrafast model systems, FeCO5 and CrCO6. The study is organized into two parts. The experimental section, though concise, presents time-resolved valence photoelectron spectra for both molecules. There are notable differences between the spectra of FeCO5 and CrCO6, particularly in terms of the minimum binding energy and the time constants for cuts at various binding energies. Even though the time resolution of 260 fs is relatively long for this type of experiment—much longer than in some previous studies—a clear distinction is still evident. Specifically, CrCO6 shows less intensity accumulation at low binding energies compared to FeCO5, and the decay rates are faster in CrCO6.

The experimental work is complemented by a detailed simulation study that explores the dissociation dynamics of the two molecules. The simulations suggest that dissociation occurs in bursts, driven by vibrational wave packet motion. They also identify the electronic states involved in the dissociation process, distinguishing between bound and dissociative states. The authors propose that the transitions from the initially excited bound states to dissociative states are the rate-limiting step for dissociation. The faster dissociation observed in CrCO6 compared to FeCO5 is attributed to the denser electronic states in CrCO6, which facilitate these transitions.

Overall, the paper is well-written, and the message is clear. However, the time resolution of 260 fs is not sufficient to fully resolve the dynamics experimentally, and it is much longer than the current state in similar experiments with the HHG sources. The trajectory simulations in Fig. 3 indicate that a resolution closer to 50 fs would be more appropriate, and it could in principle be reached. The theoretical discussion is somewhat disconnected from the experimental results, likely due to this limitation. While the authors may not be able to address this issue, I would still recommend publishing the paper in this journal, as the study of these prototype molecules is valuable.

We appreciate this criticism from the reviewer. A time-resolution of 50 fs would be much more appropriate to capture the necessary electronic and structural changes in the excited state. Due to

this limitation, we have to rely in the present study on the theoretical simulations to provide and explanation of the excited state dynamics. As reviewer 1 points out, such experiments are complex and we hope to improve the temporal resolution in forthcoming investigations.

The lack of signal in the binding energy range of 5.5-6 eV for FeCO5 is not sufficiently discussed. This is a prominent feature that should be addressed.

We thank the reviewer for pointing this out. The lack of signal in this region is due to the long lived excited state dynamics that occurs in $Fe(CO)_5$ relative to $Cr(CO)_6$. Population of states that live longer in $Fe(CO)_5$ compared to $Cr(CO)_6$ contribute to binding energies below 5 eV, which is where intensity accumulates accordingly. Once the delayed dissociation dynamics occur, we find that the binding energy shifts to higher energies indicative of the dissociation on the lower ^{1}MC states, in both cases. We hope that this clarifies the misunderstanding where large parts of our discussion aim at explaining that observation.

What was the excitation fluence, and what is the expected molecular excitation density? Did the authors conduct a UV power titration?

Explicit changes to the manuscript: We thank the reviewer very much for spotting this shortcoming. This is important important information that we now added to the experimental details in the Supporting Information in the form as follows (we did the expected power titration for $Fe(CO)_5$ in our former study in ref. 11 of the main text):

"UV excitation

The employed pulse energies for the 266 nm pump pulses amounted to 0.5 μJ/pulse with a spot size (round spot) on the sample of 100 μm in diameter (FWHM). This corresponds to a pump fluence of 6.4 mJ/cm^2. This is the same fluence we had used in our previous investigation on $Fe(CO)_5$ in ref. 11 of the main text. As shown there, this is well within the linear regime where pump-probe signals vary linearly with varying pump pulse energy (see Figure 3 in ref. 11 in the main text). And, as described ref. 11 of the main text, this fluence corresponds to an estimated excitation fraction of 6%."

The data in Figure 3 of ref. 11 of the main manuscript clearly shows that nonlinearities occur at much higher pump fluence than the one employed here.

I suggest using the terms "shift to higher/lower binding energies" instead of "red" or "blue."

We agree with the reviewer that this terminology is confusing given the extensive use of blue/red color in the figures. We have changed the text to reflect the change in this notation.

Explicit changes to the manuscript: All instances of blue/red shift have been changed accordingly.

On page 9, last paragraph: How can the maximum be at -60 fs when the time zero was previously calibrated with another sample?

Yes, this is a very good point, and it is a shame that we did not spot this ourselves. We are sorry for the confusion this may have created. We think that we mixed up baselines as our references for zero intensity (dashed lines in Figure 1f) when estimating the intensities in the binding energy regions at 5-6 eV for the different time delays. Correct use of baselines shoes that the maximum is somewhere between 0 and 20 fs. We note that this correction does not affect our conclusions.

Explicit changes to the manuscript: The text on page 6 now reads: "In $Cr(CO)_6$, in contrast (Fig. 1f), we see much weaker intensity accumulations, here at 5-6 eV and with a maximum around or shortly after 0 fs."

The authors should justify the comparison of delay traces at the same binding energy, as mentioned at the bottom of page 11. The vertical IP differs by only 0.2 eV, which justifies the comparison. However, if the IP differed by one bin size, the dynamics in adjacent bins should be compared.

We fully agree with the reviewer that this was an inconsistency. Plotting the binding energy regions for excited states when the ground-state peaks are shifted and when the same photon energy is used to excited the systems can create artefacts in the comparison. We chose to change the integration ranges for $Cr(CO)_6$ by shifting them by 0.2 eV compared to $Fe(CO)_5$ and corresponding to the shifts of the ground-state peaks of the two systems. Remaining differences in integration regions (order of 0.04 eV at maximum) are due to the detector resolution in our experiment (fundamental bin width of the experiment).

Explicit changes to the manuscript: New figure 4 and adjustment of the discussion on page 10 which now reads "Notably, when comparing the delay traces of $Fe(CO)_5$ and $Cr(CO)_6$ taken for the same binding energy intervals with respect to the respective ground-state photoelectron peaks in Fig. 2a and b (the intervals of the two systems differ by the same amount as their ground-state peaks), we can..."

It would be helpful if the authors discussed the optically allowed transitions to states S1-S11. Clearly, the transitions to the red-dissociative states are not optically allowed.

We appreciate the suggestion from the reviewer to clarify this point. All of the dynamics both experimentally and theoretically begin in an optically bright ^{1}MLCT state. The transitions to the lower ^{1}MC states are not allowed by symmetry. From a theoretical point of view, the UV spectrum of $Cr(CO)_6$ has been benchmarked by Crespo-Otero, R. and Barbatti, M. *J. Chem. Phys.* **2011**, *134* (16), 164305. and of $Fe(CO)_5$ by Banerjee, A. *et al. Nat. Commun.* **2022** *13,* 1337.

Explicit changes to the manuscript: We have made explicit statements on page 4 to describe which states are directly populated by the UV excitation.

Figure 4 would benefit from a legend explaining the different colors.

We have made an addition to Figure 4 in the main text to include a legend for the color scheme used. We have additionally added a figure in the supporting information file (Figure S8) which shows the individual-state colors rather than the grouping of states used in Figure 4.

Explicit changes to the manuscript: Figure 4 has been updated to include a legend (additional changes were made to Figure 4, see response to reviewer 1). A figure in the supporting information file has been added to have a detailed legend (Figure S8).
